# Gene expression patterns associated with influenza A and type I hypersensitivity in childhood appendicitis

**DOI:** 10.1007/s12519-026-01067-w

**Published:** 2026-08-05

**Authors:** Marc Reismann, Natalie Kiss, Moritz Markel, Kathrin Hauptmann, Jan F. Svensson, Tomas Wester, Steven W. Warmann

**Affiliations:** 1 Department of Pediatric Surgery, Charité—Universitätsmedizin Berlin, Corporate Member of Freie Universität Berlin and Humboldt Universität Berlin, Berlin, Germany; 2 Department of Pediatric Surgery, Dr. von Hauner Children’s Hospital, LMU Medical Center, Munich, Germany; 3 Institute of Pathology, Charité—Universitätsmedizin Berlin, Corporate Member of Freie Universität Berlin and Humboldt Universität Berlin, Berlin, Germany; 4 Department of Pediatric Surgery, Department of Women’s and Children’s Health, Karolinska University Hospital, Karolinska Institutet, Stockholm, Sweden

**Keywords:** Acute appendicitis, Gene expression analysis, Influenza A virus, Interferon-induced proteins, Pediatric, Type I hypersensitivity

## Abstract

**Background:**

Both influenza A infection (IAV) and type I hypersensitivity mechanisms have been independently linked to acute appendicitis. Particularly the expression of type I hypersensitivity associated with cytokine interleukin (IL) 13 has been shown to be of significance in both, appendicitis and influenza infection. The aim of the current study was to analyze possible associations of respective gene expressions at the level of mathematical correlations.

**Methods:**

We analyzed messenger RNA (mRNA) gene expressions of IAV-associated markers, hypersensitivity type I-related cytokines and inflammatory markers IL-17A and c-reactive protein (CRP) in peripheral blood mononuclear cells from 29 children aged 7–17 years, who were operated for histologically confirmed appendicitis at Charité–Universitätsmedizin Berlin between April and August 2019. Statistical relationships between gene expressions were investigated using Spearman’s correlation analysis, with a correlation coefficient *r* ≥ 0.5 or *r* ≤ − 0.5 representing high, and *r* ≥ 0.7 or *r* ≤ − 0.7 very high correlations. Statistical significance was assumed at *P* < 0.01.

**Results:**

A total of 29 patients were involved in this study. Particularly the expression of IL-13 showed highly significant negative correlations with that of IAV-specific antiviral response genes, ranging from – 0.89 (*P* < 0.0001) to – 0.49 (*P* = 0.006). A strong inflammatory background was demonstrated by very high correlations of IL-13 with CRP (*r* = 0.91, *P* < 0.0001) and IL-17A (*r* = 0.82, *P* < 0.0001).

**Conclusion:**

These findings suggest a connection between IAV infection and acute appendicitis in children, implicating sequential immune responses, including hypersensitivity type I mechanisms, in appendicitis pathophysiology.

**Graphical abstract:**

**Figure:**
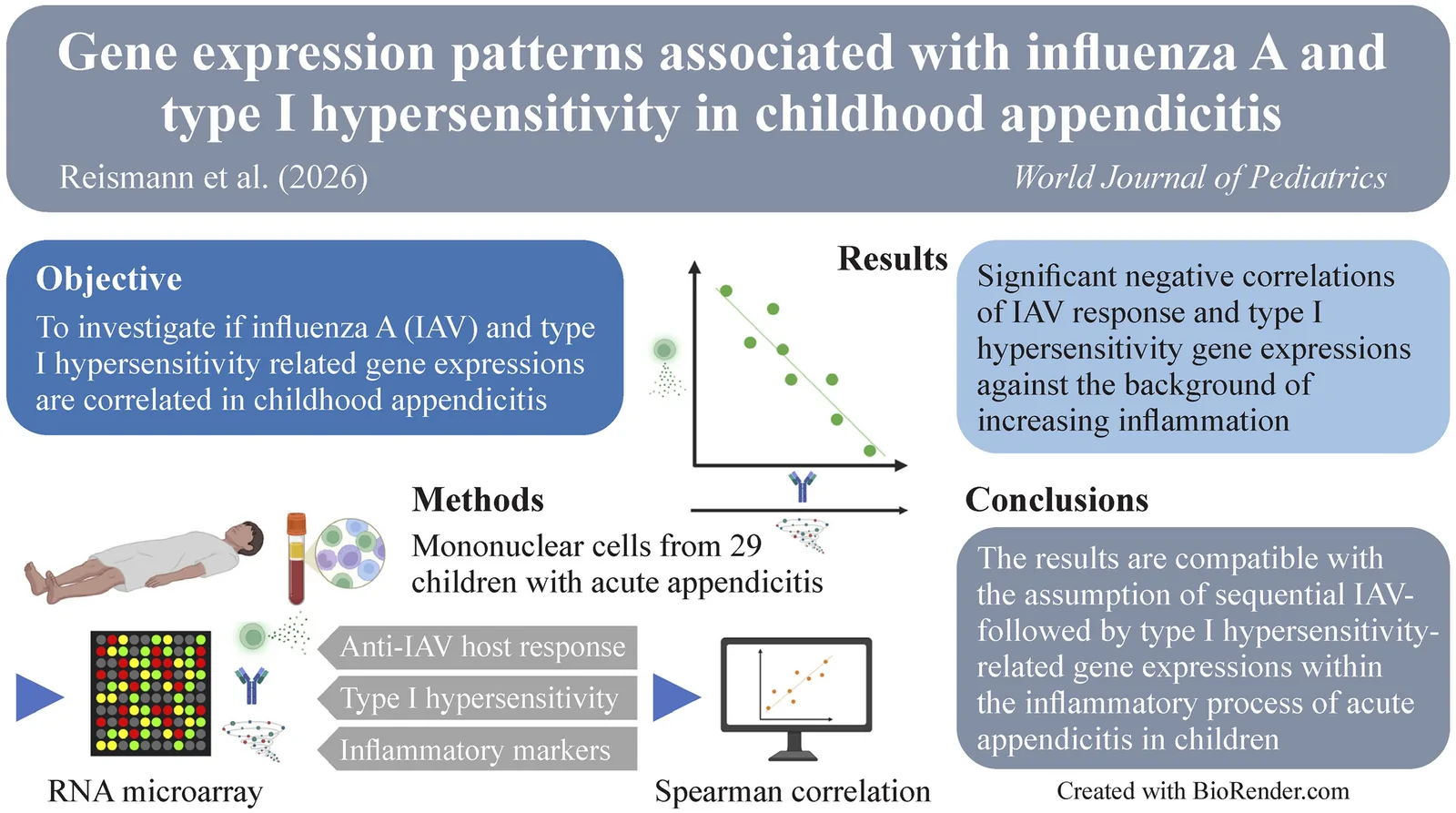

## Introduction

The possible role of influenza A virus (IAV), a single-stranded RNA (ssRNA) virus, as a cause of acute appendicitis remains a subject of debate [1]. The hypothesis is supported by numerous observations and reports over the past 120 years, which suggest a potential causative relationship between IAV infection and acute appendicitis in both children and adults [2–4]. While gastrointestinal symptoms in patients with influenza can range from mild issues, such as vomiting and diarrhea to severe, even fatal, outcomes, a definitive link between acute appendicitis and influenza virus infection remains elusive, as does the precise cause of appendicitis itself [5]. Nevertheless, a causal relationship between appendicitis and IAV remains plausible, as influenza virus has been detected in fecal samples, and its ability to replicate in the intestinal tract independently of the respiratory tract has been demonstrated, where swabs are usually taken [6, 7].

Previous studies have shown that gene expression analysis of virus-associated molecular pathways, followed by correlation analysis, is valuable for elucidating pathophysiological mechanisms, as seen during the coronavirus disease 2019 (COVID-19) pandemic [8]. Influenza infection induces a distinct antiviral gene expression response that can be differentiated from healthy individuals with high specificity [9]. This study is based on the hypothesis of a causal relationship of IAV infection and acute appendicitis in children and adolescents. The main objective was to determine whether the analysis of host gene expression profiles in children with appendiceal inflammation is compatible with this hypothesis. We conducted a correlation analysis between IAV-associated gene expressions and IAV-specific reference gene expressions in peripheral blood mononuclear cells (PBMCs).

One of the currently discussed alternative pathophysiological associations is that of a role of hypersensitivity type I, respectively T helper 2 (Th2) cell-dependent immunological mechanisms in the development of acute appendicitis [10, 11]. To this end, we included expression data of Th2-related cytokines for the investigation of associations of these two apparently independent rationales for the disease. As the secretion of Th2-related cytokines expressions in IVA infections can alternatively be related to group 2 innate lymphoid cells (ILC2), particular attention was paid to respective associations [12, 13].

## Methods

This prospective pilot study included children and adolescents aged 7–17 years who presented with clinical signs of acute appendicitis in the pediatric emergency department of Charité–Universitätsmedizin Berlin, between April and August 2019. Ethical approval was provided by our institutional ethics committee (reference number: ES2/130/16). To provide additional information on the inflammatory state, we included measurements of c-reactive protein (CRP) and interleukin-17A (IL-17A) gene expression, as both markers have been shown to correlate with the severity of appendicitis and influenza [14–17].

After obtaining written informed consent from the parents and patients, an appendectomy with subsequent histopathological examination was performed. Patients with previous conservative treatment for acute appendicitis, concomitant disease, any antibiotic treatment in the previous two weeks and an RNA integrity number (RIN) less than eight were excluded. The time between blood sample collection and isolation of PBMCs was less than one hour. Sex, age (years), and duration from the onset of symptoms until sample collection (hours) were documented.

The laboratory workflow has been published previously [18]. In short, after RNA-isolation using a NucleoSpin RNA Plus Kit (Macherey-Nagel, Düren, Germany), fluorescent complementary RNA (cRNA) was generated with the Low Input QuickAmp Labeling Kit (Agilent Technologies, Santa Clara, CA, USA). Feature Extraction Software V11 (Agilent Technologies) was used to obtain microarray data, according to the GE1_1105_Oct12 protocol. During histopathological examination, the type of inflammation was classified according to Carr’s classification [19]. Phlegmonous appendicitis is characterized by transmural infiltration of the appendix by neutrophilic granulocytes, serositis, and microabscesses. Gangrenous appendicitis is defined by the presence of ischemic areas in the appendix with transmural myonecrosis and, in some cases, eventual perforation. The nonparametric Spearman correlation coefficient (PCC), *r*, was calculated as a robust measure of the rank correlation between two variables. The coefficient can take values between − 1 and 1. Two-sided testing was performed throughout the analysis.

Following the classification made by Cohen the respective distinctions were made [20]: *r* ≥ 0.1 or *r* < – 0.1 represented low correlation; *r* ≥ 0.3 or *r* ≤ – 0.3 represented moderate correlation; *r* ≥ 0.5 or *r* ≤ – 0.5 represented high correlation; *r* ≥ 0.7 or *r* ≤ – 0.7 represented very high correlation. The level of significance was set at *P* < 0.01. Statistical analyses were performed using the GraphPad Prism software (version 11.0. f, La Jolla, CA). Heatmaps were created with the open-source application “heatmapper2” [21]. Based on the available patient data an a priori power calculation was performed according to Cohen [22]. Assuming an alpha-level of 0.01 and a power of 0.8, the case number (*n*) of 29 would be sufficient to detect statistical differences at a Spearman correlation (*r*) of at least 0.58. Correlation values in the text were rounded. Exclusion of values was not performed.

### Gene expressions

**Fig. 1:**
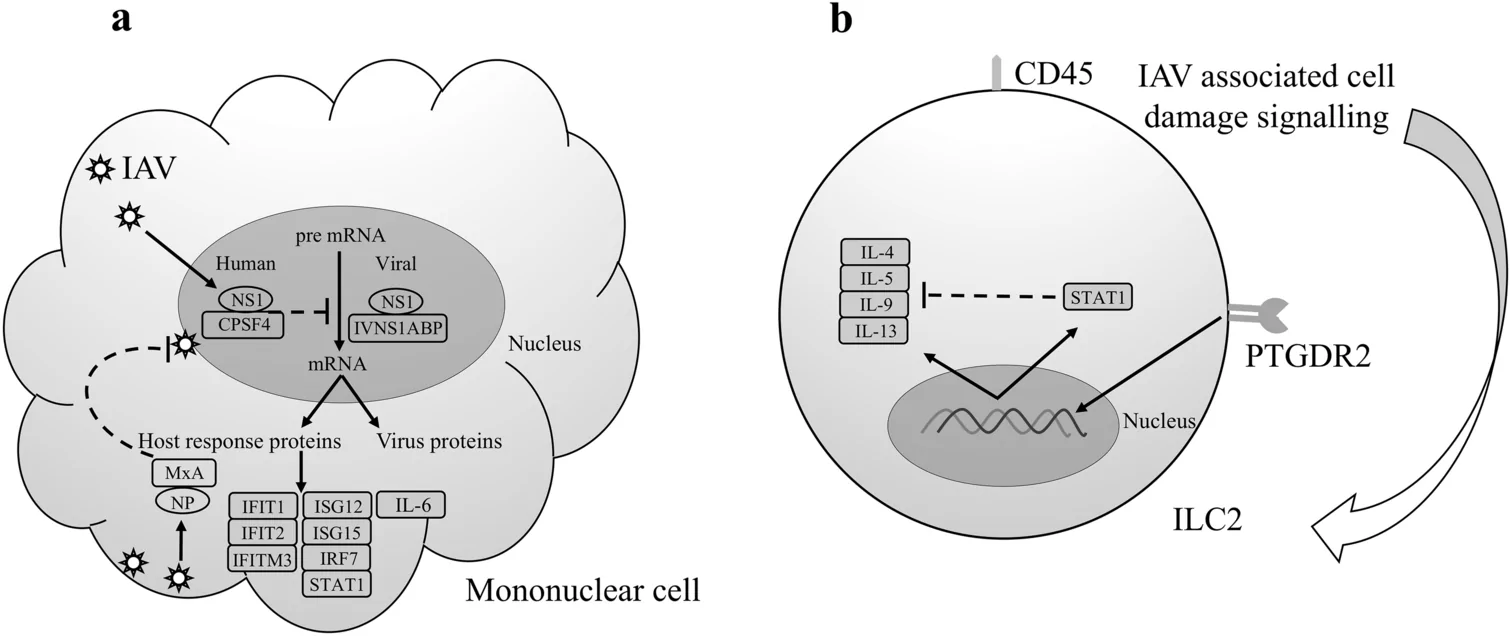
Illustration of analyzed gene expressions. **a** IVNS1ABP, CPSF4 and MxA interact directly with IAV proteins NS1 and NP [23–27]. IFIT1, IFIT2, IFITM3, ISG12 and ISG15 are particularly upregulated within IAV infections as a result of interferon production [28–31]. IRF7 and STAT1 represent transcription factors within viral infections and are highly associated with IAV infections [29]. The cytokine IL-6 has high significance in IAV infection [32]. **b** IAV infection leads to accumulation of ILC2 cells, characterized by PTGDR2 and CD45 expressions with subsequent expression of Th2 cytokines. Interleukins 4, 5, 9, 13 and immunoglobulin E are upregulated in influenza infection and acute appendicitis [12, 13]. ILC2 activity is under control of STAT1 [33]. *IVNS1ABP* influenza virus NS1A-binding protein, *CPSF4* cleavage and polyadenylation specificity factor 4, *MxA* myxovirus resistance protein A, *IAV* influenza A infection, *NS1* nonstructural protein 1, *NP* nucleoprotein, *IFIT2* interferon-induced protein with tetratricopeptide repeats 2, *IFITM3* interferon-induced transmembrane protein 3, *ISG* interferon-stimulated gene, *IRF7* interferon-related gene 7, *STAT1* signal transducer and activator of transcription 1, *IL-6* interleukin-6, *ILC2* group 2 innate lymphoid cells, *PTGDR2* prostaglandin D2 receptor 2, *CD45* cluster of differentiation 45, *Th2* T helper 2

Analyzed gene expressions with respective abbreviations are summarized in Table 1 and illustrated in Fig. 1. Influenza virus NS1A-binding protein (IVNS1ABP), cleavage and polyadenylation specificity factor 4 (CPSF4) and myxovirus resistance protein A (MxA) are characterized by direct interaction with IAV proteins nonstructural protein 1 (NS1) and nucleoprotein (NP). CPSF4 plays a crucial role in the cellular response to influenza virus infection, particularly through its interactions with the influenza viral non-structural protein NS1, a virulence factor which enhances viral replication and inhibits host immune responses [23, 24]. IVNS1ABP serves crucial roles in influenza infections, primarily through its involvement in the host cell’s response to viral presence and its interactions with viral proteins. IVNS1ABP has been shown to bind with the NS1, promoting the expression of viral genes and supporting viral RNA replication within infected cells [25]. MxA serves as a critical antiviral factor against influenza viruses, particularly through its interactions with the viral NP. This binding prevents the import of viral components into the nucleus [26, 27]. Interferon-induced protein with tetratricopeptide repeats (IFIT) 1, IFIT2, interferon-induced transmembrane protein 3 (IFITM3), interferon-stimulated gene (ISG) 12 and ISG15 are generally upregulated within viral infections as a result of interferon production and are specifically highly associated with IAV infection [28–31]. Interferon-related gene 7 (IRF7) and signal transducer and activator of transcription 1 (STAT1) represent transcription factors within viral infections and are highly associated with IAV infections [29]. IL-6 is a cytokine representing an acute phase protein with high significance in IAV infection [32]. Tissue damage and repair signaling within IAV infection leads to the accumulation of ILC2 cells, characterized by prostaglandin D2 receptor 2 (PTGDR2) and cluster of differentiation 45 (CD45) expressions, leading to subsequent expression of Th2 cytokines. Respective interleukins 4, 5, 9, 13 and immunoglobulin E (IgE) have been demonstrated to be upregulated in both, influenza infection and acute appendicitis [12, 13]. ILC2 activity is under the control of STAT1 expression [33]. CRP and IL-17A expressions have been shown to correlate with the severity of both, appendicitis and influenza [14–17].

**Table 1:** Analyzed gene expressions

Full term	Abbreviation
Influenza A virus-associated gene expressions	
Cleavage and polyadenylation specificity factor 4	CPSF4
Influenza virus NS1A-binding protein	IVNS1ABP
Myxovirus resistance protein A	MxA
Interferon-induced protein with tetratricopeptide repeats 1	IFIT1
Interferon-induced protein with tetratricopeptide repeats 2	IFIT2
Interferon-induced transmembrane protein 3	IFITM3
Interferon-stimulated gene 12	ISG12/IFI27
Interferon stimulated gene 15	ISG15
Interferon-related gene 7	IRF7
Signal transducer and activator of transcription 1	STAT1
Interleukin-6	IL-6
Type I hypersensitivity related gene expressions	
Interleukin-4	IL-4
Interleukin-5	IL-5
Interleukin-9	IL-9
Interleukin-13	IL-13
Immunoglobulin E	IgE
Group 2 innate lymphoid cell (ILC2) marker gene expressions	
Prostaglandin D2 receptor 2	PTGDR2
Cluster of differentiation 45	CD45
Inflammatory marker gene expressions	
Interleukin-17A	IL-17A
C-reactive protein	CRP

## Results

### Basic characteristics of participants

In the study period from April to August 2019, respiratory influenza infection rates were particularly low after the end of the seasonal flu wave [34]. Among the 33 patients with histologically confirmed appendicitis, four were excluded due to low RNA quality; ultimately, 29 patients (15 boys and 14 girls) were analyzed. Mean age of participants was 11.3 years [standard deviation (SD): 2.6, range: 7.1–17.0]. Mean time from onset of symptoms to blood sample collection was 28.3 hours (SD: 16.9, range: 5.0–80.5). None of the patients showed typical signs of respiratory influenza infection.

### Correlations of analyzed gene expressions

The analyzed hyperresponsiveness type I-related gene expressions were generally negatively correlated with IAV-related gene expressions IFIT1, IFIT2, IFITM3, IRF7, ISG12, ISG15, STAT1 and IL-6. Type I-related IL-13 showed the most negative statistically significant correlations, ranging from – 0.89 (IL-13/STAT1, *P* < 0.0001) to – 0.49 (IL-13/IL-6, *P* = 0.006).

Other hyperresponsiveness type I-related gene expressions were negatively correlated with IAV-related expressions to a different extent. Thus, correlations of IgE expressions were significantly negative [range from – 0.59 (IgE/IFIT1, *P* = 0.0008) to – 0.48 (IgE/IFITM3, *P* = 0.0081)], followed by correlations with IL-4 [range from – 0.54 (IL-4/IFIT1, *P* = 0.0023) to – 0.37 (IL-4/IFIT2, *P* = 0.0479)]. IL-9 expressions were moderately negatively correlated with hyperresponsiveness type I-related gene expressions [range from – 0.43 (IL-9/IFIT1, *P* = 0.0197) to – 0.37 (IL-9/IFIT2, *P* = 0.0509)], followed by IL-5 [range from – 0.01 (IL-5/IFIT1, *P* = 0.945) to 0.06 (IL-5/STAT1, *P* = 0.753)].

Within IAV-related gene expression, all correlations, but one were statistically significant, ranging from 0.42 (IFIT2/IL-6, *P* = 0.0229) to 0.94 (IFIT1/STAT1, *P* < 0.0001). Correlations within hyperresponsiveness I-related gene expressions were inconsistently statistically significant, ranging from – 0.23 (IL-5/IL-9, *P* = 0.0225) to 0.58 (IL13/IgE, *P* = 0.0011).

Hyperresponsiveness type I-related gene expressions showed neutral up to very high correlations with CRP, ranging from – 0.08 (IL-5/CRP, *P* = 0.6976) to 0.91 (IL-13/CRP, *P* < 0.0001), while correlations between CRP and IAV-related gene expressions were essentially significantly negative, ranging from – 0.87 (IVNS1ABP/CRP, *P* < 0.0001) to – 0.51 (IL-6/CRP, *P* = 0.0048). Correlations of hyperresponsiveness type I- and IAV gene-related expressions with IL-17A gene expressions showed a similar picture [range from 0.18 (IL-5/IL-17A, *P* = 0.339) to 0.82 (IL-13/IL-17A, *P* < 0.0001), and range from – 0.90 (IFIT1/IL-17A, *P* < 0.0001) to – 0.62 (IL-6/IL-17A, *P* = 0.0004)].

**Fig. 2:**
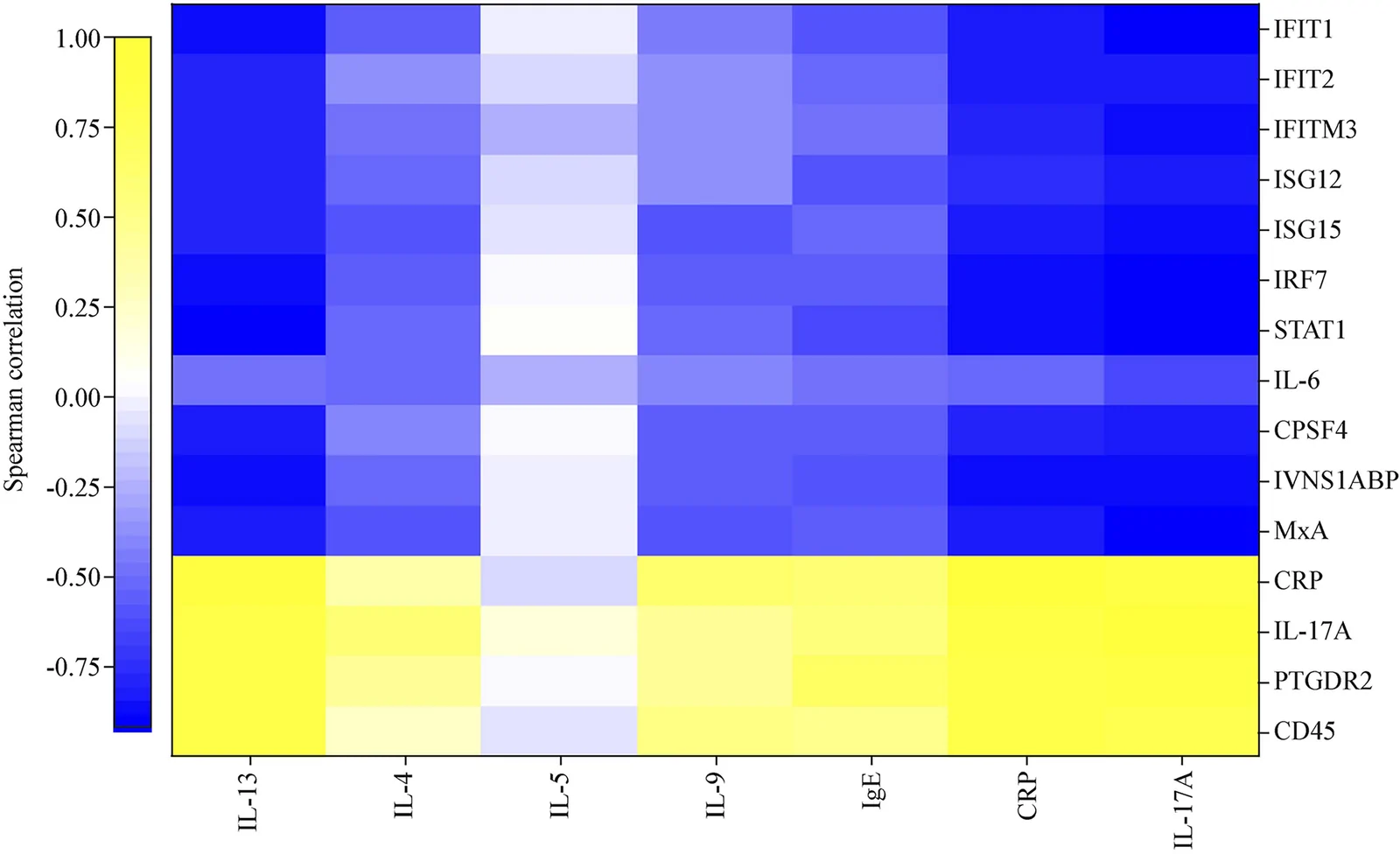
Heatmap demonstrating the Spearman correlation coefficients between the analyzed gene expressions. *IVNS1ABP* influenza virus NS1A-binding protein, *CPSF4* cleavage and polyadenylation specificity factor 4, *MxA* myxovirus resistance protein A, *IAV* influenza A infection, *NS1* nonstructural protein 1, *NP* nucleoprotein, *IFIT2* interferon-induced protein with tetratricopeptide repeats 2, *IFITM3* interferon-induced transmembrane protein 3, *ISG* interferon-stimulated gene, *IRF7* interferon-related gene 7, *STAT1* signal transducer and activator of transcription 1, *IL-6* interleukin-6, *ILC2* group 2 innate lymphoid cells, *PTGDR2* prostaglandin D2 receptor 2, *CD45* cluster of differentiation 45, *Th2* T helper 2. Color scheme: yellow: positive correlation; blue: negative correlation; white: neutral

Hyperresponsiveness type I-related gene expressions showed neutral up to very high and statistically significant correlations with PTGDR2 gene expressions, ranging from 0.03 (IL-5/PDGDR2, *P* = 0.861) to 0.84 (IL-13/PTGDR5, *P* < 0.0001), while IAV-related gene expressions were essentially highly significantly negatively correlated with PTGDR2, ranging – 0.87 (IFIT1/PDGDR2, *P* < 0.0001) to – 0.68 (IL-6/PTGDR2, *P* < 0.0001). Correlations with CD45 showed a similar picture. Hyperresponsiveness type I-related gene expressions showed neutral up to very high and statistically significant correlations with CD45, ranging from – 0.06 (IL-5/CD45, *P* = 0.7395) to 0.83 (IL-13/CD45, *P* < 0.0001), while IAV-related gene expressions were essentially highly significantly negatively correlated with CD45, ranging from – 0.90 (CPSF4/CD45, *P* < 0.0001) to – 0.50 (IL-6/CD45, *P* = 0.006). Correlations of PTGDR2 and CD45 with inflammatory CRP and IL-17A gene expressions were very high (range: 0.77–0.87, *P* < 0.0001). The detailed results are demonstrated in Fig. 2.

### Temporal correlations of analyzed gene expressions

There were no significant correlations of any of the investigated gene expressions and the time from the onset of symptoms to sample collection (*P* > 0.010).

## Discussion

The associations between influenza virus infections and appendicitis have been suggested primarily based on epidemiological observations over longer periods and certain numerical relationships between these two diseases in the context of IAV outbreaks and pandemics, as well as on case reports [1–4, 35]. The derived hypothesis on a causal relationship between influenza and appendicitis might not appear obvious. Influenza is primarily expected to cause respiratory symptoms, which are not regularly found in patients with appendicitis. However, influenza infection regularly goes along with severe gastrointestinal symptoms, and anorexia, diarrhea, vomiting, and abdominal pain are common manifestations [5]. Indeed, IAV has the capacity for intestinal infiltration and replication independent of the respiratory tract [6, 7].

The vermiform appendix is possibly particularly affected by viral infections. Thus, according to the so-called safe-house theory, the appendix protects commensal intestinal bacteria located in its lumen from viral infections to allow the subsequent bacterial recolonization of the intestine [36]. Although IAV has been detected in inflamed appendices by antibody staining and polymerase chain reaction (PCR), a distinct causative relation could not be demonstrated, because a variety of other alternative viruses has been found [1]. This might underscore the function of the vermiform appendix as local “virus sieve”, but limits the possibilities for detection of a specific, potentially causative virus.

This study is performed against the background of another influential theory on the emergence of acute appendicitis. Findings on increased numbers of atopy-related eosinophils together with increased expression of associated cytokines in patients with appendicitis have led to the suggestion that allergic hyperresponsiveness type I-related mechanisms have pathophysiological significance in acute appendicitis. Particularly elevated levels of atopy-related cytokines IL-4, IL-5, IL-9, IL-13 and IgE have been found in patients with acute appendicitis [10]. However, theories on IAV and hyperresponsiveness type I as causes of acute appendicitis are not necessarily contradictive, but rather underscore the complexity of the pathophysiology. Particularly in the early phase of IAV infection, eosinophils are able to provide immediate antiviral functions [37]. Specifically, eosinophils accompanied by related cytokine production serve as antigen-presenting cells for stimulation of IAV-specific T cells [38]. Even more striking, within viral infection proinflammatory ILC2 cells are a major source of IL-4, IL-5, IL-9 and IL-13 production [12].

In IAV infection ILC2 cells are characterized by IL-13 production [12]. The findings in our study show that IL-13 seems to have a central role. The expression of the cytokine is significantly correlated with expressions of ILC2 markers PTGDR2 and CD45 and with the expression of inflammatory markers CRP and IL-17A, which is compatible with the above-mentioned IL-13-associated proinflammatory role of ILC2 cells in IAV infection. Indeed, in an analysis of IL-4, IL-9, IL-13 and IgE in children with acute appendicitis, only IL-13 was found to be associated with severity of the disease, which is similar to our data [39].

The observed negative correlations between IAV-associated gene expression and the expression of IL-13 and IL-17A may be explained by the temporal dynamics of the immune response. Interferon-associated gene expressions constitute the first line of defense against viruses, peaking within the initial hours after infection and subsequently declining as specific cellular cytokine responses emerge [40]. Thus, the duration of symptoms as reported by patients may not accurately reflect these molecular changes over time.

Based on the application of a robust correlation analysis, an integrative model for the hypothetical role of IAV infection in the pathophysiology of acute appendicitis in children can be formulated. Our findings are compatible with the hypothesis that the expressions of hyperresponsiveness type I-related cytokines in children with acute appendicitis are associated with IAV-related gene expression patterns against the background of increasing inflammation. These results are further supported by the observed correlations involving IL-13, which has previously been demonstrated in both the pathophysiology of appendicitis and IAV infection.

The strengths of the study are particularly given by the prospective design of the study based on a sound sample size after proper power calculation, allowing the application of the robust Spearman correlation analysis. The correct classification of the patients was ensured by a very careful histopathological assessment of the resected appendices. The results are backed by independent previous evidence on gene expressions in patients with acute appendicitis and influenza infection.

This study still has several limitations. As a pilot study, it did not include a healthy control group, following consultation with the ethics committee, and the inclusion of inflammatory parameters only partially compensates for this limitation. Additionally, virological confirmation of virus presence (e.g., by PCR) was not performed, as previous attempts at direct virus detection in intestinal material have been hampered by mixed infections and the inability to identify a single causative virus. Throat swabs might be unsuitable for detecting intestinal infections, as the infectious routes of intestinal IAV infection are by far not clarified [7]. Different infectious routes might explain the lack of seasonal relationship of intestinal and respiratory influenza infection [35]. Future studies should incorporate reliable methods for direct virus detection or assess IAV presence through specific cellular immune response assays, such as antigen-specific proliferation assays with tissue-resident T cells. Finally, as a correlation analysis, this study cannot establish causality or confirm active infection.

In conclusion, the study results demonstrate significant mathematical correlations between IAV-related and type I hyperresponsiveness-associated gene expressions in children with histopathologically verified acute appendicitis in children. This takes place against a strong pro-inflammatory background. However, definitive proof of a causal relationship is still pending. The proposed integrative model of IAV- and hyperreactivity type I-related mechanisms should, therefore, give rise to particularly combined virological and functional-immunological investigations involving cells of the appendix tissue to gain further insights into possible causal relationships.

## Data Availability

The datasets analyzed during the study are available in the Open Science Framework repository (www.odf.io) under the URL https://osf.io/xwnr3/.
